# The impact of chest radiography and Xpert MTB/RIF testing among household contacts in Chennai, India

**DOI:** 10.1371/journal.pone.0241203

**Published:** 2020-11-04

**Authors:** Ramya Ananthakrishnan, Rajeswaran Thiagesan, Sheela Auguesteen, Nalini Karunakaran, Lavanya Jayabal, Jagadeesan M, Robert Stevens, Andrew Codlin, Jacob Creswell

**Affiliations:** 1 REACH – Resource Group for Education and Advocacy for Community Health, Chennai, Tamil Nadu, India; 2 GCC RNTCP – Greater Chennai Corporation Revised National Tuberculosis Control Programme Chennai, Tamil Nadu, India; 3 Independent Consultant, Manchester, United Kingdom; 4 Stop TB Partnership, TB REACH, Geneva, Switzerland; The University of Georgia, UNITED STATES

## Abstract

Tuberculosis prevalence surveys have demonstrated the benefit of screening with chest x-ray (CXR) and sensitive diagnostic tests compared to symptoms and smear microscopy. However, in programmatic practice there is little evidence on the yield of different algorithms. We implemented contact tracing in Chennai, India for adult sputum-positive TB patients registered from January 2015 to March 2016. Patients with symptoms or abnormal X-ray findings further underwent testing using Xpert MTB/RIF (Xpert) and smear microscopy. A retrospective cohort study was done to summarize the key findings. We verbally screened 5553 contacts for symptoms, CXR through private sector collaboration, Xpert, and smear microscopy. Overall, 1312 (23.6%) contacts screened positive. CXR alone identified 531 (40.5%) of them, 679 (51.8%) were symptom-positive only, while 102 (7.8%) were positive on both the symptom and CXR screen. Overall, 35 bacteriologically positive cases were identified (0.7%). A standard approach of symptoms screening followed by microscopy identified only 9 (25.7%) of the total number of bacteriologically positive cases, whereas the combination of a CRX screening followed by microscopy identified 13 (37.1%) of the cases. The algorithm of symptoms screening followed by Xpert testing, detected 20 cases, whereas the combination of symptoms and CXR followed by Xpert increased this number to 35 (75% increase compared to symptoms and Xpert). Optimal use of more sensitive screening tests, better diagnostic tests, and novel private sector engagement can improve diagnostic yield in a programmatic setting.

## Introduction

India has the highest TB burden in the world, with a quarter of world’s cases and has the largest number of people with TB who are currently missed [1]. Most people with TB who are missed by the Revised National Tuberculosis Control Programme (RNTCP), are thought to seek care in the vast private sector and are not reported [2]. TB prevalence surveys throughout Asia have shown that a large proportion of people who have bacteriologically confirmed TB do not complain of symptoms. These people are only detected through other screening methods like chest x-ray (CXR) and the use of more sensitive diagnostic tests [3]. For decades, the standard approach to case detection has been wait for people who are ill to present to public facilities complaining of cough and test them using smear microcopy [4]. Recently, there has been a large focus globally on different approaches that reach more people who are sick with TB [5–8]. The World Health Organization (WHO) has several guidelines that relate to improving TB case detection including screening household contact investigation, private sector collaboration, screening with CXR, and testing with Xpert MTB/RIF (Xpert) [9]. While Xpert has been well established as a more sensitive test than smear microcopy [10], few studies have looked at the value of combined screening approaches settings outside of prevalence surveys. A couple recent studies showing the value of CXR screening over symptom screening in clinic or hospital settings [11, 12] and one in contact investigation [13], but the data is limited.

One of most recognized and embraced approaches to enhance TB case detection has been through the investigation of household contacts (HHC) of people with TB, who have much higher rates of TB than the general population [14]. Yet implementation of contact investigation is often deficient [15]. In India, the RNTCP recommends screening of all HHC of smear-positive pulmonary tuberculosis cases for symptoms and if the screen positive, diagnostic testing. Nevertheless, only a small proportion of HHC are screened, and the approach is generally based on symptom screening followed by smear microscopy [16].

We present the results of an evaluation of systematic screening of household contacts using both CXR and symptom questions as screening tools coupled with standard and molecular diagnostics in an urban population in Chennai, India. The screening and testing were done as part of an innovative Public-Private NGO Partnership model.

## Methods

The intervention was conducted from January 2015 through March 2016 in Chennai, India (population of 7.2 million). At the time of the study, the RNTCP in Chennai was implemented through 36 Tuberculosis Units (TU) distributed among 15 administrative zones, with each TU catering to ~200,000 population. The study was initially implemented in 15 TUs, covering approximately ~ 4.7 million of the population. Towards the end of 2015, these 15 TUs were converted into 36 TUs covering approximately ~ 7.2 million population. All adult (>15) sputum-positive TB patients identified from the RNTCP TB registers were considered index cases and were eligible for inclusion in the study. Field officers met each registered sputum smear-positive (SS+) patient within a month of starting anti-TB treatment and provided counselling and education on contact investigation and chemoprophylaxis. After obtaining written informed consent, the field officer collected details of index patient’s HHC on a tablet based electronic register. HHC were defined as any person sleeping in the index patient’s home for more than 7 days during the last 3 months. The index patient was provided with one coupon for each HHC enumerated, and instructed to provide the coupons to their HHC if they wished to invite them to participate. This coupon allowed the HHC to receive symptom screening as well as a free CXR at a network of private sector labs. To facilitate the screening of HHC, a travel allowance (~1.7 USD) was provided for those who participated. Through a combination of phone follow-ups and house visits, multiple attempts were made to encourage HHC screening.

Since access to CXR in public facilities is often difficult, distances can be far, and most people seek private sector care initially, an agreement with a chain of private labs for CXR services was negotiated prior to the study. The digital CXR was provided free to the HHC and later reimbursed by the study team at a low cost (~3 USD). At the private screening facilities, health workers were present and administered a simple verbal screening tool (questionnaire) to ascertain cough of any duration and the presence of other TB symptoms including fever, weight loss, hemoptysis, night sweats, and swelling in neck, armpits or groin. Women of reproductive age (15–44) were additionally screened verbally to ascertain their pregnancy status. Subsequently, all HHCs received a digital CXR which was read by two trained radiologists and graded abnormal or normal.

If the HHCs reported any of the symptoms or showed radiographic abnormalities, or both, they were counselled to provide sputum for diagnostic testing and given a falcon tube to collect an early morning sample. The HHC was requested to bring their sputum sample to the nearest TU facility. From the TU facilities, the samples were transported for testing by Xpert and smear microscopy by the National Institute for Research in Tuberculosis (NIRT). Those HHCs who were not able to produce sputum or had extra-pulmonary symptoms were connected to the Medical Officer of the TU for further management.

All HHC diagnosed with TB were registered for treatment in the respective TU as per RNTCP guidelines. All data in the field was captured via Android based tablets using Epi Info, a free public health data capture application from the US Centers for Disease Control and Prevention. Data from the tablets was synced monthly into the main database where descriptive statistics were calculated, and analysis was conducted using IBM SPSS 16. The main outcomes of interest were the yield of different combinations of screening and testing (symptom and CXR screening and smear and Xpert testing). The number needed to screen (NNS) and number needed to test (NNT) were calculated for each screening and testing algorithm (using symptoms and/or CXR and using microscopy or Xpert). Bacteriologically positive (B+) TB was defined as having a positive smear or Xpert result. Permission to conduct the study was provided by the Chennai Corporation and ethical clearance for the study was obtained from the REACH Independent Ethics Committee. Written consent was obtained from all the participants in the study.

## Results

**Fig 1:**
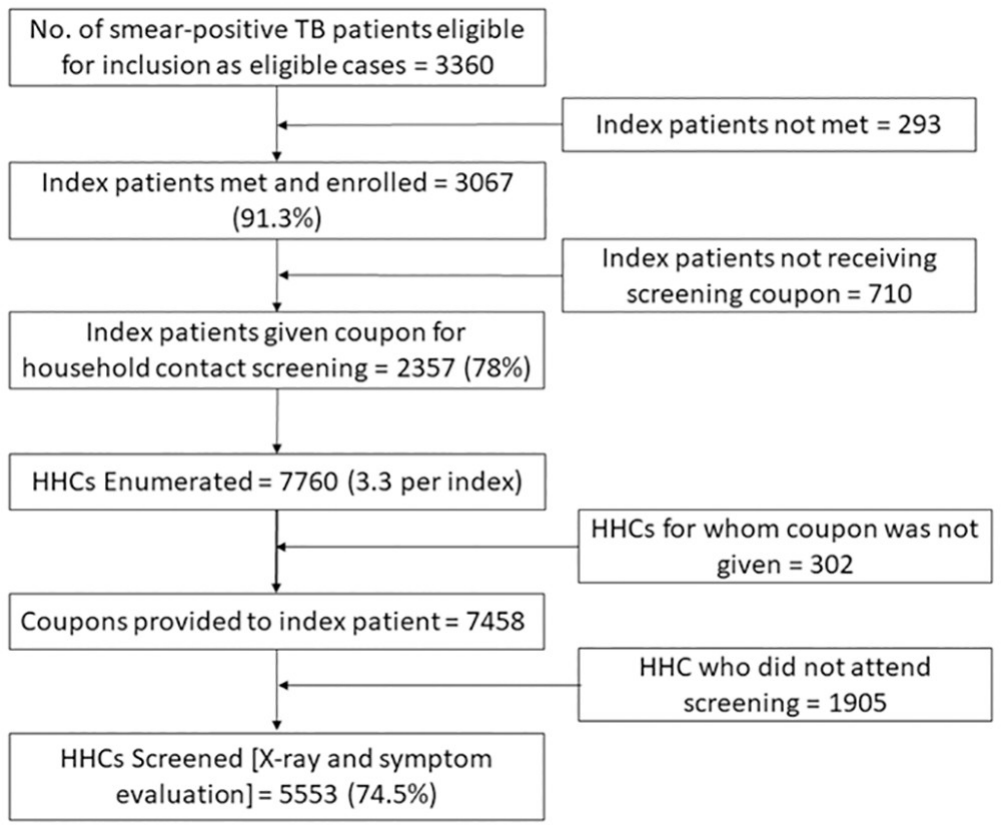
Flow chart on contact screening process.

A flow diagram showing study enrollment is shown in Fig 1. A total of 3,784 new SS+ patients were registered in the intervention area during the study period. Of them, 3,360 (88.8%) were eligible for inclusion in the study. We were not able to contact 293 (8.7%) of eligible index cases meaning that we attempted to enroll 3,067 index cases (91.3%) in the study. Reasons for not being able to contact the index case included the inability to follow-up within the time frame (49%), pre-treatment loss to follow up (10%), transfer to treatment in the private sector or alternate treatment methods (10%), transfer out to other DOTS center (9%), migration (13%), in patient admission of the index case (3%) and died before contact (6%). Of the 3,067 index patients contacted, 408 (13.3%) lived alone and not included in the study while 171 (5.6%) declined to participate. The final sample included 2,357 index cases who were provided coupons for their HHCs. 79% of the index TB patients were males and 86% were between 15 and 59 years old. 73% of them were had no prior TB history. The number of HHCs enumerated from the initial index case interviews was 7,760 however 302 were not given coupons as per the index case instructions so we distributed 7,458 screening coupons. During the study period 5.553 (74.5% of people with coupons) completed the screening process. We identified 47 HHCs who had already been diagnosed with TB through the standard RNTCP algorithm of symptom screen followed by smear microscopy. These HHCs were not screened further. Of the HHCs attending the private labs for screening, 72.8% were older than 14 and 31.8% were male. The median age of screened contacts was 30.

**Fig 2:**
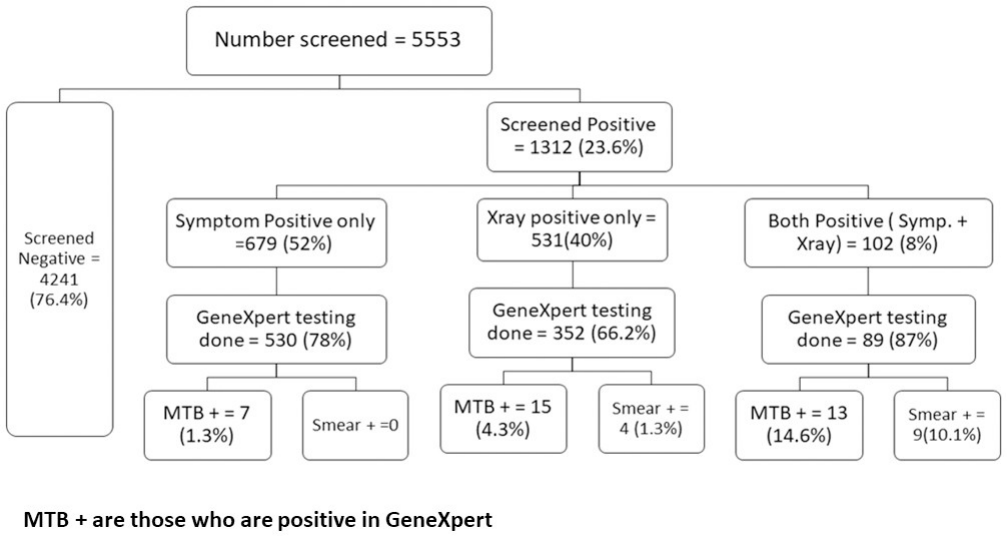
Flow chart on no. of household contacts screened by various methods and their yield.

Fig 2 shows the results of the evaluation of the screening and testing algorithms. The CXR and symptom screening resulted in 4,241 HHC (76.4%) with no TB-related symptoms and normal CXR findings leaving 1,312 (23.6%) who screened positive. Of them, 679 (51.8%) were symptom-positive only, while 102 (7.8%) were positive on both the symptom and CXR screen, and 531 (40.5%) were only positive on the CXR screen. Sputum samples were collected from 971 (74%) of those who screened positive. There were differences in the proportion of people producing sputum across the different groups. Among HHC with both symptom and CXR positive screens, 89 (87%) were able to provide a specimen for testing while 352 (66.2%) of those with only CXR abnormalities could do so. After testing with Xpert and smear microscopy, we identified 35 B+ TB cases, all of whom were positive on Xpert. Overall, smear microscopy detected 13 (37.1%) of the Xpert positive cases. An additional five individuals (12.5% of all TB cases identified) were diagnosed with TB after clinical evaluation and initiated on anti-TB treatment among whom three had an abnormal CXR and two were diagnosed with extra pulmonary TB.

### Diagnostic tests and algorithms

Overall, 5,553 people were screened, and using CXR and symptom screening together resulted in 971 people being tested to identify 35 B+ cases. Using symptom screening alone resulted in 619 HHC who produced a sample for diagnostic testing, and Xpert detected 20 cases (NNS of 278 and NNT of 31). However, using microscopy after symptom screening identified less than half (n = 9) of these individuals (Table 1). The routine screening and diagnostic algorithm (symptom screening followed by microscopy) therefore detected 26% of all B+ cases among HHC and had the highest NNS (619) of the different algorithms tested. When CXR and symptoms screening were combined, and Xpert was used, the number of B+ cases increased by 75% (35 cases compared to 20 with only symptoms). Among the 35 B+ contacts, 56% were males and 86% were between 15 and 59 years old.

**Table 1:** Number of household contacts needed to screen and test to identify a bacteriologically positive TB case.

Strategy/Algorithm	Screened	Tested	NNS	NNT	Number of TB Cases	% of all B+ TB
**Symptoms only**						
Microscopy	5553	619	617	69	9	26
Xpert	5553	619	278	31	20	57
**X-ray only**						
Microscopy	5553	441	427	34	13	37
Xpert	5553	441	198	16	28	80
**Symptoms and X-ray**						
Microscopy	5553	971	427	75	13	37
Xpert	5553	971	159	28	35	100

NNS = Number Needed to Screen (Number of people needed to screen to diagnose a bacteriologically positive pulmonary case, who has not previously been diagnosed by the health system).

NNT = Number Needed to Test (Number of people needed to test to diagnose a bacteriologically positive pulmonary case, who has not previously been diagnosed by the health system).

TB = Tuberculosis.

B+ = Bacteriologically Positive.

CXR alone as a screening tool identified 441 HHC who produced a sample for testing and detected 28 of the 35 B+ cases (80%) if Xpert was used, as seven other B+ cases had symptoms but a normal CXR reading. Similar to symptom screening, among the 28 cases screening positive on the CXR, microscopy alone would have detected less than half (n = 13) of them. Of the 530 HHC tested with a normal CXR but who were symptomatic 7 B+ cases were identified (1.3% positivity), and none of them were identified by microscopy (Fig 2). Smear microscopy performed best among HHC with both CXR and symptom screens positive; of the 13 B+ cases detected, nine were smear-positive, but still missed 4/13 B+ cases. Using CXR only followed by Xpert produced the lowest NNT (16) but missed seven B+ cases. Using CXR and symptoms followed by Xpert produced the lowest NNS (159) and had the second lowest NNT (28) while identifying all 35 B+ cases.

We identified 40 HHCs with TB (35 B+) through the screening and diagnostic testing process, while 47 others were identified as already being diagnosed through routine care. Therefore, a total of 87 HHCs were diagnosed with TB. Overall, the prevalence of TB among HHC was 1477/100,000 (1.4%) while our screening of HHC found a prevalence of 720/100,000 (0.7%).

## Discussion

While TB prevalence surveys have shown the impact of using CXR screening and more sensitive diagnostic tests to identify more people with TB, there have been few studies to do so as part of programmatic delivery. We found that adding CXR to the standard symptom screen increased the proportion of people detected with B+ TB by 75% (35 cases instead of 20) when Xpert testing was used as the diagnostic test. In addition, we showed that a high proportion of HHC can be screened with CXR even without dedicated facilities by the use of innovative private partnerships.

While TB prevalence surveys can detect the greatest proportion of B+ TB among adults in a given population, they are not feasible as largescale TB active case finding approaches due to the complicated logistics and high implementation costs [17]. Prevalence surveys have documented that 40–79% of people with TB did not report symptoms and were only identified through CXR screening. Often, more than 50% of bacteriologically confirmed cases are sputum smear-negative [3]. Our results in a contact investigation study were similar and suggest that when feasible, CXR screening combined with more sensitive diagnostic testing will improve the diagnostic yield in other interventions to improve case detection.

Conducting symptom screening is quick, and inexpensive and can be done by a lay worker with basic training [18] but our results suggest it misses many people who have B+ TB disease among HHC. A study among HHC from South Africa found that smear microscopy was even less efficient in detecting culture-confirmed TB, and that most HHC did not report symptoms in a high HIV-burden setting [19]. The South African study did not evaluate CXR, and instead tested all HHC who could produce sputum. In Chennai, a low HIV prevalence setting, CXR and symptom screening could eliminate the need for diagnostic testing for three quarters of HHC identified. The overall yield of 1.4% we identified is less than a study done in the same setting (5.3%) [20] Possible reasons for this could be a larger sample size of HHCs and our project was done under programmatic conditions rather than a clinical trial. An analysis of different approaches to HHC tracing in 19 projects demonstrated a large range of findings from 0.1% to 6.2% using different criteria for screening and testing highlighting the heterogeneity of results that can be documented [21]. In many settings, the old standards of passive case finding from earlier TB strategies are still being used by TB programs—where cough screening is done to identify people for further diagnostic testing, primarily with smear microscopy. CXR was shunned as part of the early global strategies for TB control [22], but there has been a large interest more recently to use CXR as a screening tool which can help identify more people who need testing, [11] and reduce the need for more expensive diagnostic tests [23, 24]. While CXR accessibility may be limited and can cost up to 20 USD in some high burden countries [25], in many Asian settings it is widely available, relatively inexpensive and used extensively in the private sector [8, 26]. While a plethora of studies have been published on different ways to improve TB case detection including improved outreach [27, 28], systematic screening in facilities [29], more sensitive diagnostics [30], strengthening public-private links [31], as well as contact investigation [20], there have been only a handful of published studies that have evaluated the impact of using CXR in addition to symptom screening for TB outside of prevalence surveys [11–13]. Two of these studies were done in high HIV burden settings. Nevertheless, our results were comparable in terms of additional TB detected using the CXR screening with Xpert and add to a small evidence base.

The proportion of HHCs screened among those receiving coupons (74.5%) was similar compared to other published studies [14, 19, 32, 33]. We believe decentralized access to CXR (usually within 2 to 3 km) and the consistent follow-up by the health staff were critical factors which positively influenced participation. A proportion of patients refused coupons as they had not disclosed their disease to their family and reinforces the importance and need for stigma reduction and community advocacy.

Our study has a number of limitations. We identified 47 HHC who had already been diagnosed with TB. We are unable to confirm if these people were in fact HHC or if they were the true ‘index case’. The identification of a large number of HHC already on treatment may point to lack of proper contact tracing as part of the routine operations. We referred people who could not produce sputum for clinical evaluation, but ideally more people with negative diagnostic tests would be clinically diagnosed. Also, we didn’t enter the details of people who were not willing to participate in the program in the database. The proportion of clinically diagnosed TB in India is 63% in 2016 [1] while we found only 12.5%. It is likely that more clinically diagnosed TB could be possible and that our results are underestimates. About one quarter of the index patients did not receive screening coupons. The main reason was more than half of them were living in single rooms without household contacts. This is commonly seen in urban metropolitan settings given migration from other districts and states. Amplifying screening of social contacts or larger contact groups could identify additional people with TB [34].

## Conclusion

India’s ambitious National Strategic Plan (2017–22) aims to achieve universal access to TB services and treatment success rates of at-least 90%, and recently Prime Minister Modi announced a plan to eliminate TB by 2025 [35]. While new tools are certainly needed, innovative use of current tools can have a major impact and allow us to better reach all people with TB. Including CXR in addition to symptom screening and the use of more sensitive diagnostic tests such as Xpert or other WHO recommended tests as well as novel private sector engagement can help reach these ambitious goals by identifying people with TB who otherwise will be missed.
